# Tumor-derived small extracellular vesicles in cancer invasion and metastasis: molecular mechanisms, and clinical significance

**DOI:** 10.1186/s12943-024-01932-0

**Published:** 2024-01-19

**Authors:** Chi Zhang, Chaoying Qin, Saikat Dewanjee, Hiranmoy Bhattacharya, Pratik Chakraborty, Niraj Kumar Jha, Moumita Gangopadhyay, Saurabh Kumar Jha, Qing Liu

**Affiliations:** 1 Department of Neurosurgery, Xiangya Hospital, Central South University, Changsha, 410008 China; 2 The Institute of Skull Base Surgery and Neuro-Oncology at Hunan Province, Changsha, 410008 China; 3 Advanced Pharmacognosy Research Laboratory, Department of Pharmaceutical Technology, Jadavpur University, Kolkata, 700032 West Bengal India; 4 Centre of Research Impact and Outreach, Chitkara University Institute of Engineering and Technology, Chitkara University, Punjab, India; 5 Department of Biotechnology, School of Applied & Life Sciences (SALS), Uttaranchal University, Dehradun, 248007 India; 6 Department of Biotechnology, School of Life Science and Biotechnology, Adamas University, Barasat, Kolkata, 700126 West Bengal India; 7 Department of Zoology, Kalindi College, University of Delhi, New Delhi, Delhi, 110008 India

**Keywords:** Biomarkers, Cancer, Epithelial-mesenchymal transition, Metastasis, MicroRNA, Oncogenic transformation, Tumor-derived small extracellular vesicles, Tumor microenvironment

## Abstract

The production and release of tumor-derived small extracellular vesicles (TDSEVs) from cancerous cells play a pivotal role in the propagation of cancer, through genetic and biological communication with healthy cells. TDSEVs are known to orchestrate the invasion-metastasis cascade via diverse pathways. Regulation of early metastasis processes, pre-metastatic niche formation, immune system regulation, angiogenesis initiation, extracellular matrix (ECM) remodeling, immune modulation, and epithelial-mesenchymal transition (EMT) are among the pathways regulated by TDSEVs. MicroRNAs (miRs) carried within TDSEVs play a pivotal role as a double-edged sword and can either promote metastasis or inhibit cancer progression. TDSEVs can serve as excellent markers for early detection of tumors, and tumor metastases. From a therapeutic point of view, the risk of cancer metastasis may be reduced by limiting the production of TDSEVs from tumor cells. On the other hand, TDSEVs represent a promising approach for in vivo delivery of therapeutic cargo to tumor cells. The present review article discusses the recent developments and the current views of TDSEVs in the field of cancer research and clinical applications.

## Introduction

Extracellular vesicles (EVs) are heterogeneous set of membrane-bound vesicles present in body fluids e.g. blood, urine, saliva, and ascites. Small extracellular vesicles (SEVs) are EVs that are less than 200 nm in diameter and play vital roles in various physiological and pathological states [1]. Exosomes and ectosomes are SEVs produced by normal and tumoral cells and released into the extracellular microenvironment. They contain a diverse range of biomolecules, including lipids, proteins, and nucleic acids, and associate themselves with a variety of biological functions. These entities play a central role in modulating immune response, promoting tumorigenesis, facilitating tumor invasion, and initiation of metastasis [2]. Tumor-derived SEVs (TDSEVs) can govern the development of tumors and metastasis by controlling multiple types of cells, including but not limited to immune cells, endothelial cells and epithelial cells. SEVs facilitate the process of tumor angiogenesis, promote infiltration of tumor cells and disrupt tight junctions formed by endothelial cells allowing tumor cells to escape from bloodstream and invade other organs [3]. Meanwhile, SEVs can selectively target epithelial cells, the extracellular matrix (ECM) thereby facilitating the metastatic cascade [4]. The primary manifestation of this influence is readily seen in the occurrence of EMT.

SEVs have been the subject of persistent investigations due to their potential as molecular cargoes [5]. Recent investigations have underscored the importance of TDSEVs in the initiation process, development, and dissemination of cancers [6]. TDSEVs are efficacious molecular carriers that transport a diverse range of over 40,000 proteins, including actin, tubulin, actin-binding molecules, cytosolic proteins, proteins originating from plasma and endosomal membranes etc. [7, 8]. While constituents like heat shock proteins Hsp60, Hsp70, Hsp90, CD63, ESCRT machinery, and cytoskeletal elements are ubiquitously shared by SEVs, distinct proteins like MHC Class I and II exhibit specificity corresponding to the cellular source of the vesicles [9]. High levels of Hsp90 in cancer cells are essential to counteract various stress like hypoxia, acidosis, and metabolic and nutrient deficiency, thus the presence of Hsp90 in exosomes may play important roles in pre-tumoral niches [10]. The tetraspanin family of proteins assumes essential functions in orchestrating the complex regulatory mechanisms regulating cancer cell migration and the interplay between cancer cells and endothelial cells. The presence of high levels of tetraspanins in SEVs is a widely acknowledged phenomenon, and targeting tetraspanin holds significant clinical potential. Along these lines, several in vitro and in vivo studies have reported that tetraspanins CD9 and CD82, impede interaction with integrins limiting invasion and migration of cancerous cells [11, 12]. Since tetraspanins form specialized trans-membranal platforms, specific targeting may offer therapeutic opportunities as demonstrated by the targeting of CD81 in preclinical models of triple-negative breast cancer (TNBC) [13].

In addition to proteins, SEVs transport lipids, mRNA, and DNA, all of which can potentially initiate malignant alterations and promote cancer progression [14]. miRs are key functional components of SEVs, but their composition displays an irregular profile in cancer cells, SEV miRs can function as genetic messengers, influencing gene expression patterns of recipient cells [15]. Finally, it should be noted that long non-coding RNAs are also key components of the molecular cargo and can interfere with gene expression by targeting miRs or by modifying histone complexes.

In 2020, the number of cancer-related fatalities was nearly 10 million, and this figure is projected to rise to over 13 million by 2030 [16]. SEVs, membrane-bound vesicles with low immunogenicity, hold considerable therapeutic potential to fight against cancer. Certain SEVs produced by immune cells aid in tumor cell eradication [17]. Furthermore, chemotherapeutic agents targeting oncogenic pathways could be incorporated into SEVs to perform anti-tumor functions. Conversely, directing interventions towards TDSEVs also holds promise as a strategy to hinder cancer metastasis. This involves impeding the dissemination of biologically active constituents from the tumor to distant organs. Present review describes the underlying mechanisms of SEV formation, their roles in cancer invasion, metastasis, immunological regulation, and their impact on the tumor microenvironment. Herein, the diagnostic, prognostic, and therapeutic features of TDSEVs are critically discussed along with the potential challenges concerning recent findings and future research.

## TDSEVs: generation and features

The identification of SEVs as the mediator of vesicle-based intercellular communication has transformed cancer metastasis research. The vesicles exhibit a size range of about 30–150 nm with a density range of around 1.08–1.19 g/ml [18]. Interestingly, EVs derived from tumor cells do not always comply to this size range and are often found to be much larger in size. Cancer cells produce an increased number of EVs compared to normal cells, with the ability to activate a variety of functional responses [19, 20]. According to the International Society for Extracellular Vesicles, EV is described as the generic term for particles naturally released from the cells, that are cup-shaped and delimited by a lipid bilayer and cannot replicate, as they are devoid of a functional nucleus [1]. A single cell can release EVs of different size, density, subcellular origin, and function. As a result, heterogeneity is a key property of EVs. Because of the difficulties in determining the biogenesis of an EV, size is one of the commonly utilized operational factors to mark a vesicle. On the basis of size, EVs are classified as SEVs and medium/large EVs based on their diameter, with size ranges of roughly 100–200 nm (small), and more than 200 nm (medium and/or large) [1].

Small membrane crinkles within the endocytic system coincide with the formation of several microdomains where the cargoes concentrate. Endosomes, integral to the endocytic process are membraned cell organelles found in all eukaryotic cells. Early endosomes (EE) are formed by inward budding of plasma membranes. EEs either fuse with the plasma membrane or go to lysosomes where the cargo gets destroyed. As part of the endocytic system, EEs convert into late endosomes (LE) whereby intraluminal vesicles (ILVs) are formed in endosomes by inward budding (several cellular components such as nucleic acids, proteins etc. are entrapped within ILVs). The assembly of the ESCRT-machinery begins with the localization of the ESCRT-0 on EEs. PI3P helps in the recruitment of the early ESCRT protein Hrs. Hrs then forms a complex with Tsg101 to include ESCRT-I in the process [21]. ESCRT-I then binds to ESCRT-II. The subsequent binding of CHMP6 of ESCRT-III with ESCRT-II activates CHMP4 at the endosomal membrane [22]. CHMP4 and ubiquitinated protein play pivotal roles in the inward budding of the membrane to produce ILVs [22]. An endosome containing many ILVs are known as multivesicular bodies (MVBs). If the content is destined for degradation, these MVBs can merge with the lysosome, or else they can fuse with the cellular membrane, releasing the ILVs as EVs into the extracellular environment through vesicular secretion [23–25]. GAP TBC1D15 inactivates Rab7 and removes the same from MVBs/LEs upon being recruited by Arl8b/SKIP/HOPS. MVBs/LEs then migrate towards the cell's periphery through the action of kinesin motors [26]. Rab31 also engages GAP TBC1D2B to inactivate Rab7, preventing MVB degradation while enhancing MVB fusion with the plasma membrane to facilitate exosome secretion [27]. Intracellular transport of MVBs is facilitated by molecular motors that traverse the cytoskeleton and microtubule networks, ultimately leading to their arrival at the plasma membrane. ESCRT machinery, which consists of four ESCRT complexes (ESCRT-0, -I, -II, and -III), is the canonical sorting apparatus MVBs are moved by molecular motors along the cytoskeleton and microtubule networks to the plasma membrane. During ILV maturation, exosomal mechanisms selectively gather cargo components. Thus far, alternative mechanisms that do not rely on ESCRT, such as those involving ceramide, tetraspanins (CD63), and Rabs (Rab31), have been postulated to explain ILV generation in ESCRT-depleted cells [28]. ESCRT-independent processes, depend largely on complex lipids and other protein-related pathways [29]. In neutral sphingomyelinase 2 (nSMase2)-ceramide pathway, the enzyme nSMase2 is essential for the conversion of sphingomyelin to ceramide. FAN, a WD-repeat protein, boosts nSMase2 activity to increase ceramide synthesis [30]. Phosphatidylethanolamine-conjugated LC3 on MVBs, recruits FAN to the limiting membrane in many cancer cells. Following that, cargoes with LC3-interaction regions are incorporated into MVBs via the nSMase2-ceramide-dependent pathway [31]. Ceramide is a complex lipid that can self-associate to form raft-like formations and initiate membrane curvatures with regard to inward budding to generate ILVs [32]. Caveolin-1 is an integral membrane protein with a hairpin-like structure that binds cholesterol on the membrane which also sorts exosomal cargo in a ESCRT-independent manner but majorly limited by the nSMase2-ceramide pathway [33]. Flotillins, a group of membrane scaffolding proteins aid in forming lipid rafts, and are involved in diverse biological activities such as endosome trafficking and protein sorting [34]. Flotillns and tetraspanins also help in cargo sorting of ILVs. CD63 in conjunction with Apolipoprotein E, for example, stimulates ILV formation and aids in ILV sorting of PMEL via both ESCRT-dependent and ceramide-dependent mechanisms. CD63 knockdown or knockout consistently lowers ILV development and exosome biogenesis [35]. There are few other ESCRT-independent mechanisms of exosome biogenesis as well, however complete understanding is yet to be achieved.

**Fig. 1:**
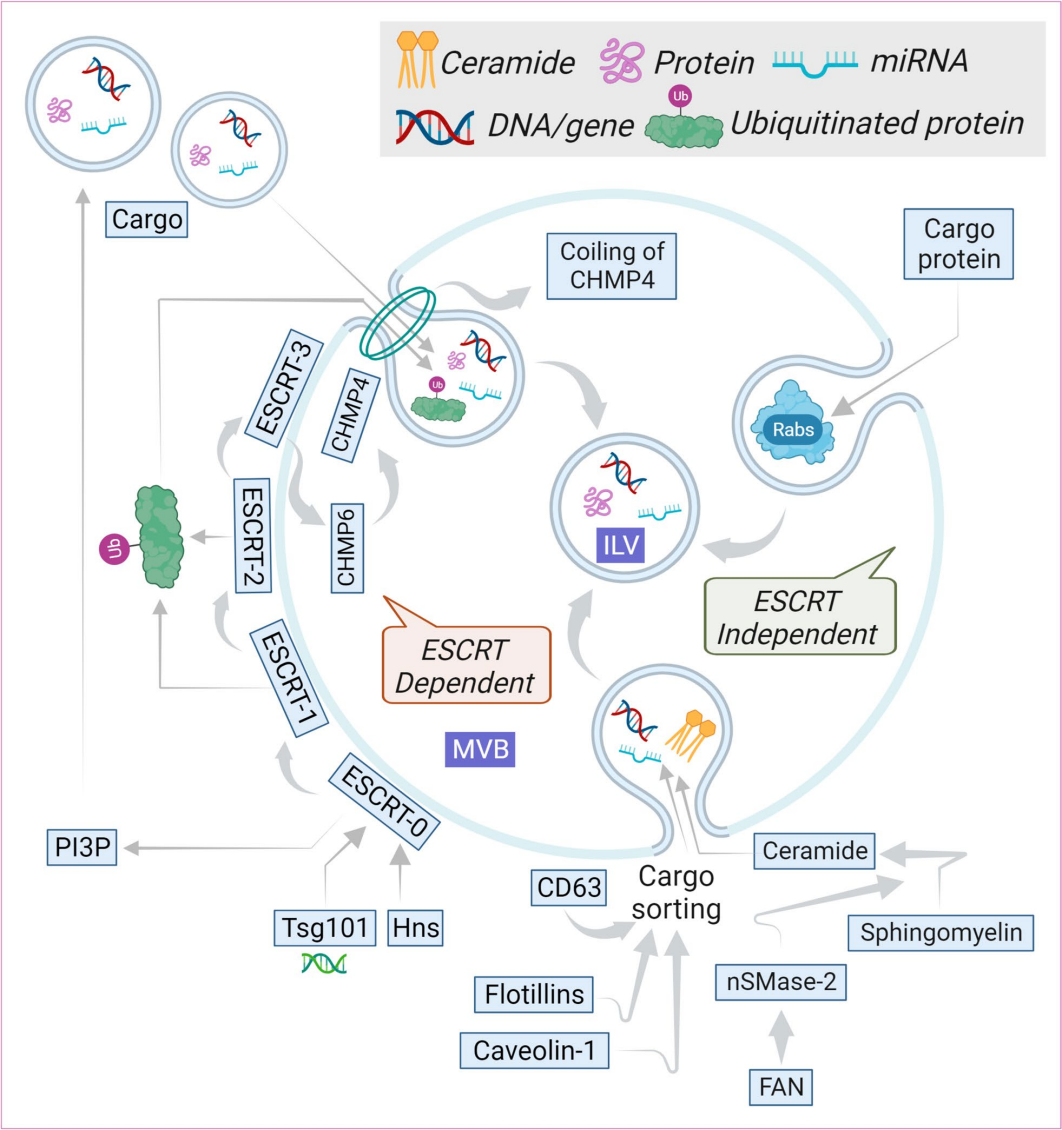
Schematic representation of biogenesis of ILVs. ILVs are the precursors of exosomes created by the inward budding of microdomains and their fission. Assembly of ESCRT machinery starts with the localization of ESCRT-0 on EEs. PI3P aids in recruiting early ESCRT protein Hrs. Hrs then binds with Tsg101 to involve ESCRT-I in the process. ESCRT-I, in turn, binds with ESCRT-II. Subsequent binding of CHMP6 of ESCRT-III with ESCRT-II activates CHMP4 to the endosomal membrane. CHMP4, along with ubiquitinated protein play a pivot in the inward budding of the membrane to form ILVs containing proteins, DNA and miRs. Polymerization of CHMP4 forming spiral coils store potential energy, on elastic compression, this energy gets released giving rise to negative curvature within the membrane As part of the endocytic mechanism, exosome precursors are discharged into MVBs. Also, ESCRT-independent mechanisms involving ceramide, tetraspanins (CD63), and Rabs (Rab31), respectively, have been proposed. In the nSMase2-ceramide pathway, FAN upregulates ceramide production from sphingomyelin. Ceramide, self-associates to form raft structures within the cell membrane to initiate the formation of curvature and subsequent budding. Caveolin-1 and flotilin play vital roles in raft formation and sorting activities. After the sorting procedures are finished, MVBs actively bypass lysosomal fusion. Rabs ensure to prevent MVBs from degradation before fusing with the plasma membrane. Sphingomyelin tends to reorganize the plasma membrane into lipid raft microdomains which subsequently trigger negative curvature in the membrane. Arrows indicate downstream cellular events. EE, early endosome; ESCRT, an endosomal sorting complex required for transport; ILVs, intraluminal vesicles; miR, microRNA; MVB, multivesicular body

The initial step towards the release of exosomes from intracellular compartments into the extracellular environment involves the merging of multi-vesicular endosomes with the cellular surface and/or retrograde budding from plasma membrane, resulting in its unique protein-lipid makeup. The enhanced release of exosomes from cancer cells is primarily associated with the overexpression of Rab3D, a member of the RAS oncogene family (Rab3D), activation of transduction pathways such as the Wnt pathway, and the presence of an acidic microenvironment that facilitates cell fusion events [36]. Following the completion of sorting procedures, MVBs exhibit active avoidance of fusion with lysosomes. Production of ILVs in MVBs is depicted in Fig. 1.

Ectosomes represent a broader size-range compared to exosomes (100 nm-500 nm), and all ectosomes are not classified as SEVs [24]. In contrast to exosomes, little is known about ectosome biosynthesis. Exosomes are generated through the endosomal complex following the fusion of MVBs with plasma membrane, whereas ectosomes/ microvesicles originate from budding of the plasma membrane. The formation of ectosomes necessitates the accumulation of payloads at the cytosolic surface of specialized microdomains of the plasma membrane. Concurrent membrane dynamics entail the outward budding and fission of the respective microdomains. Interestingly, this phenomenon could be attributed to the rearrangement of the asymmetric layers of membrane phospholipids caused by Ca^2+^-dependent enzymes, flippases, and floppases [37]. In contrast, to the activity of a minimum of two ESCRT complexes activate mechanisms similar to those that occur during ILV production [38]. Other mechanisms regulating ectosome shedding from the plasma membrane include the small GTPase Arf6, which is involved in vesicular traffic, and the Rho family small GTPases, RhoA, Cdc42, and Rac1, acting by contracting cortical actin underneath the plasma membrane [39–42]. Ectosome production is frequently dispersed throughout numerous, broad portions of the plasma membrane exposed to diverse stimuli. Similar to exosomes, ectosome membranes contain significant quantities of cholesterol, sphingomyelin, and ceramide [41–44].

It is well established that tumor cells can release SEVs containing a variety of bioactive molecular components to message neighboring or distant cells [45, 46]. SEVs have been detected in the tumor microenvironment, and emerging evidence supports the potential roles TDSEVs in tumor growth, invasion, angiogenesis, metastasis, immune responses, and resistance to chemotherapeutic agents [9, 47–49]. The nucleic acids (non-coding RNA, mRNA, and DNA fragments) in TDSEVs participate in intercellular communication, chemotherapy resistance, micro angiogenesis, immune response control, management of the tumor microenvironment, and promotion of tumor invasion and metastasis. Additionally, membrane proteins, nuclear-related proteins, and the family of quadruplex cross-linked proteins can be expressed on TDSEVs selectively. Among them, CD9, CD63, and CD81 are frequently used for SEV screening [50]. Moreover, TDSEVs contain immunosuppressive substances that inhibit immune cell anti-tumor activity. TDSEV components can govern signal transmission between donor and recipient cells and serve as biomarkers for tumor progression and anti-tumor actions. Regulating the quantity of a specific nucleic acid or protein in TDSEVs thus provides a new route and/or target for tumor diagnosis, and treatment.

**Fig. 2:**
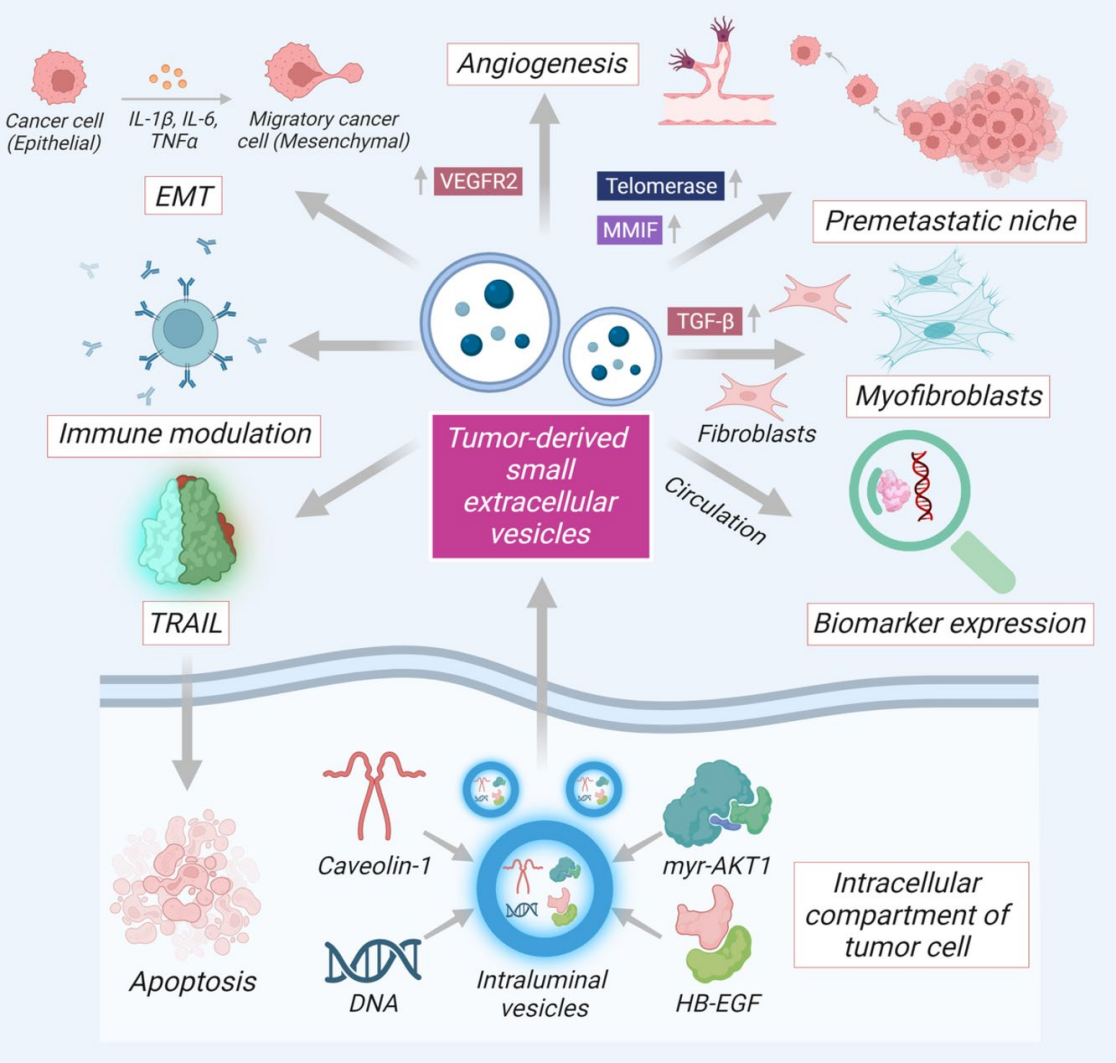
Pharmacological mechanisms and properties of TDSEVs. Components of TDSEVs participate in intercellular communication and management of tumor microenvironment. TDSEVs contain a plethora of proteins and enzymes engaged in metabolic processes. ILVs are primarily characterized by abnormal production of several different oncoproteins, including caveolin-1, HB-EGF, and MyrAkt1 TDSEVs orchestrate tumorigenic cascade via regulating early metastasis processes, pre-metastatic niche formation carrying telomerase and MMIF, immune system regulation, angiogenesis initiation, ECM remodeling, EMT pathway. TGF-β is transferred by TDSEVs from cancerous cells to healthy fibroblasts, increasing the development of myofibroblasts. DNA within TDSEVs exhibit major translational significance by regulating circulating biomarkers aiding in the early identification of cancer and metastasis. SEV miRs, can in turn lead to a pro-metastatic inflammatory response involving cytokines TNF-α and interleukins. Interestingly, TRAIL, from TDSEVs can restore apoptosis at tumor sites. ‘↑’ represents upregulation. Arrows indicate downstream cellular events/activation

TDSEVs support cancer cells to reorganize their microenvironment, enhancing the propensity for tumor initiation and dissemination. [51]. TDSEVs contain a plethora of enzymes engaged in the metabolic processing of glucose, glutamine amino acids, and instructions for extracellular communication, and differentiation and migration of cells (Fig. 2). Delivery of TDSEVs to host cells bears the potential to induce genetic instability and oncogenic mutations within target cells propelling normal cells into malignant cells [52]. Interestingly, EVs from brain cancer cells bear the ability to spread the oncogenic receptor, epidermal growth factor receptor variant III (EGFRvIII) to cancer cells not expressing this receptor, over the course of tumor progression [53]. Transforming growth factor beta (TGF-β) is transferred by TDSEVs/TDEs from cancerous cells to healthy fibroblasts, increasing the development of myofibroblasts [23, 54]. TDSEVs from cancer-associated fibroblasts, on the other hand, prevent mitochondrial oxidative phosphorylation, regulating the metabolic processes of cancer cells [55]. SEVs released from pancreatic tumors play vital roles in the formation of metastatic niches that carry macrophage migration inhibitory factor and/or telomerase [56]. Interestingly, TDSEVs containing tumor necrosis factor-related apoptosis-inducing ligand (TRAIL) have restored apoptosis at the tumor (lymphoma and melanoma) sites [57]. Clearly, TDSEVs involved in many signaling cascades possessing the capability to alter tumor microenvironment, have gained overwhelming interest among researchers.

## TDSEVs in the tumor microenvironment

Beyond genetic and epigenetic modifications within cancer cells, the intricate interplay between components of the tumor microenvironment, such as cancer cells and cancer-associated stromal cells (CASCs), assumes a multifaceted function in the oncogenesis and cancer progression [58]. CASCs can range from fibroblasts and endothelial cells to immune cells and neurons, depending on the tumor location and tissue type [59]. TDSEVs are thought to guide CASCs to adopt and function to support and nurture cancer cells [60]. However, newer research has shown that CASCs are capable of transforming into cancer cells [59]. The identification of the numerous cell and non-cell constituents of the tumor microenvironment has greatly improved our understanding of the molecular pathways involved in cancer biology. The involvement of TDSEVs in the modification of tumor microenvironment is discussed below.

### ECM remodeling

ECM is a multi-molecule network comprising of collagen, fibrin, proteoglycans, and elastin which supports cellular mechanical functions and affects the risks of tumor initiation and susceptibility to metastasis [61, 62]. Alterations in ECM are prevalent in tumor metastasis. It has been proposed that matrix metalloproteinases (MMPs) such as MMP1, MMP3, MMP9, and MMP13, particularly MMP13, can mediate ECM breakdown [63]. Furthermore, LEs can internalize membrane type 1 MMP (MT1-MMP or MMP14) via recycling endosomes [64]. LEs play a role in exosome biogenesis which may explain why exosomes contain MMPs, e.g. MMP1 is seen in EVs produced from ovarian cancer cells [65]. MT1-MMP, present within SEVs derived from tumor cells participate in the activation of pro-MMP2 and the breakdown of ECM components like collagen type I and gelatin in the adjacent cells [63]. Interestingly, non-coding RNAs found in TDSEVs can also influence ECM breakdown, for example, SEV lnc-MMP2-2 derived from lung cancer is able to modulate migration and invasion by raising MMP2 expression [66]. Earlier studies have shown that fibroblasts treated by melanoma-derived exosomes demonstrate higher cell invasion potential [67]. These events were attributed to the release of miR-21, contained into TDSEVs, resulting in upregulated expression of MMP2 and MMP9 [66, 67]. While TDSEVs can directly mediate ECM breakdown, they can also indirectly influence MMP expression. Members of the mitogen-activated protein kinase (MAPK) family, such as extracellular signal-regulated kinase (ERK) 1/2 and JNK, increase MMP2 expression [68]. Many studies have revealed that TDSEVs, such as those produced by hepatocellular carcinoma, and colorectal cancer, can increase metastasis via the ERK signaling pathway [69–71]. Caveolin-1 can influence MMP9 expression [72]. When caveolin-1 is lost in macrophages that are involved in metastasis, the activity of vascular endothelial growth factor (VEGF) A/VEGF receptor (VEGFR) 1 increases. This, in turn upregulates the production of MMP9 and colony-stimulating factor 1 (CSF1), which promote angiogenesis and growth of metastases. Though the mechanisms underpinning metastatic promotion are not fully understood, it is often accepted that ECM degradation enhanced by TDSEVs is one of the major processes leading to metastasis.

### Pre-metastatic niche formation and roles of integrins

Recent research on SEV-mediated metastasis has found a link between SEVs and pre-metastatic niches [73]. SEVs promote the establishment of pre-metastatic niches as well as secondary sites to facilitate metastasis [74]. During metastasis, a specific form of circulating tumor cells (metastasis-initiating cells) is implicated. Metastasis-initiating cells can act on other cells by secreting SEVs and by reprogramming neighboring stromal cells in order to form a more amicable tumor microenvironment. Organotropism can be determined by TDSEVs, for example, prostate cancer cell-derived SEVs have a predilection for bones because these SEVs favor cell-to-bone trafficking [75]. However, one of the largest mysteries surrounding cancer metastasis is the intricate process and molecular pathways involved in the TDSEV-mediated colonization of circulating tumor cells. TDSEVs also express certain integrin patterns, which relate to specific cell types in target to aid in predicting future pre-metastatic niches at organotropic sites, e.g. the bones, lung, liver, lung, and brain [76].

Integrins are a wide family of cell adhesion receptors made up of transmembrane glycoproteins that are involved in cell adhesion mechanisms [77]. Integrins, particularly αvβ3, have been implicated in cancer stem cell mesenchymal transition, adherence, and survival in a tumor microenvironment [78]. Furthermore, integrin β4 is able to serve as a predictive biomarker for TNBC enabling to identification of more aggressive mesenchymal carcinoma cell subtypes [79]. αvβ6, an RGD-binding protein can actively promote metastasis in case of a variety of cancers [80, 81]. Emerging evidence suggests that TDSEVs carry integrins like α6β1, α6β4, αvβ5, and αvβ3 on their surface, leading to adhesion to the target tissues resulting in metastasis [82–87]. Growing evidence indicates that exosomes from tumor cells fuse preferentially with resident cells, and this organ-specific uptake eventually prepares the pre-metastatic niche [76].

### EMT

**Fig. 3:**
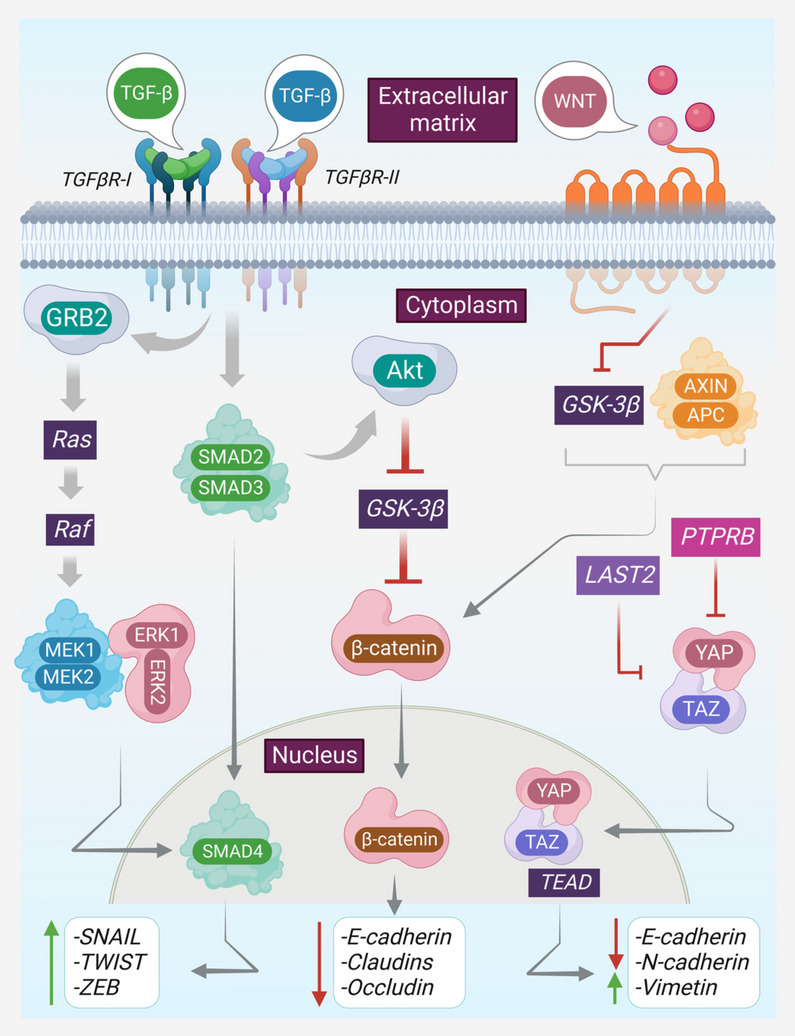
Regulation of intracellular and extracellular markers towards EMT. EMT is regulated by intracellular and extracellular markers. Wnt signaling inhibits GSK-3β which is an inhibitor of β-catenin, β-catenin in turn downregulates epithelial marker E-cadherin through influencing transcriptional factors. YAP/TAZ translocates to the nucleus and binds to TEAD to enhance the mesenchymal markers i.e. vimentin and N-cadherin and downregulate E-cadherin. TGF-β signaling can trigger the MAPK/ERK pathway and Smad pathway. Smad inhibits GSK-3β and can activate transcription factors including SNAIL, ZEB, and TWIST, resulting in a loss of cell–cell adhesion and an increase in mesenchymal markers. ERK pathway also contributes to Smad4 at the nucleus in the enhancement of the mesenchymal transcription factors.‘↑’ indicates upregulation, ‘↓’ indicates downregulation. Arrows indicate downstream cellular events/activation and lines indicate inhibition. Akt, Ak strain transforming; EMT, epithelial-mesenchymal transition; GSK-3β, glycogen synthase kinase-3 beta; PTPRB, receptor protein tyrosine phosphatase; TAZ, transcriptional coactivator with PDZ-binding motif; TEAD, transcriptional enhanced associate domain; Wnt, Wingless-related integration site; YAP, yes-associated protein; ZEB, Zinc-finger E-box-binding homeobox

During EMT, epithelial cells convert into mesenchymal cells, gaining mesenchymal characteristics that enable such cells to move into adjacent tissues and invade them [88]. Epithelial cells, in the course of actions lose E-cadherin expression, cell–cell adhesion, and apicobasal polarity while gaining vimentin expression [89]. TDSEVs play important roles in EMT to transport messages from tumors to recipient cells, resulting in alterations in recipient cell behavior and associated microenvironment (Fig. 3). Through the loss of junction and adhesion capacity mediated by proteins, DNAs, mRNAs, miRs, and lncRNAs, TDSEVs induce EMT; and epithelial cells acquire mesenchymal features becoming more susceptible to malignancy [90]. Much research has been undertaken to study the crucial role of TDSEVs to generate cancer-associated EMT. Based on the SEV-derived components involved in signaling cascades, the key components of SEVs that can serve as prospective EMT regulators are further described.

Deregulation of the Hippo pathway which primarily regulates the growth of organs can result in the widespread incidence of tumors [91]. It has been revealed that the Hippo pathway hinders tumor signaling cascades by regulating the tumor suppressor YAP/TAZ through a restriction in organ size [92]. After the Hippo pathway is inactivated, YAP/TAZ translocates to the nucleus in order to bind to TEAD [93, 94]. TEAD-regulated transcription of target gene reduces expression of epithelial markers while increasing the expression of mesenchymal markers [89]. Additionally, YAP interacts with transcription factors involved in EMT such as SNAIL, SNAIL, and ZEB1; the resulting complexes enhance cancer stem cell characteristics a hallmark of metastatic cells [95].

Several studies demonstrate that TDSEVs can upregulate EMT by altering the Hippo pathway. SEV miR-31-5p generated from tumor-associated macrophages (TAMs) can reduce the expression of the tumor suppressing gene LATS2 via disruption of the Hippo signaling cascade [96]. Furthermore, miR-665 is abundant in SEVs derived from hepatocellular carcinoma cells, where it can suppress PTPRB expression and promote EMT by lowering Hippo signaling activity [97–99]. Additionally, in gastric cancer, RP11-323N12.5 upregulates tumor growth by promoting YAP1, and higher expression of RP11-323N12.5 is detected in tumor-infiltrating leucocytes (TILs), which might be generated from SEVs and is also linked to tumor progression [100]. These findings imply that tumor-derived SEV components can target the Hippo pathway and trigger EMT during tumor cell development.

The Wnt/β-catenin pathway, one of the most well-studied mechanisms leading to EMT is frequently linked to TDSEVs [100]. SEV miR-34a-5p from oral squamous cell carcinoma promotes EMT via Ak strain transforming (AKT)/glycogen synthase kinase-3 beta (GSK-3β)/β-catenin/SNAIL signaling [101]. Hypoxia-induced hepatocellular carcinoma cells can produce SEV miR-1273f, promoting EMT in normoxia-conditioned hepatocellular carcinoma cells by triggering the Wnt/β-catenin cascade [102]. The lncRNA TIRY works as a miR sponge, decreasing miR-14 expression and promoting EMT during oral cancer [103]. The Wnt/β-catenin cascade is also triggered by colorectal cancer cell-derived SEV circABCC1, although the underlying molecular processes have not been studied [104]. Aside from non-coding RNAs, many Wnt ligands are supplied through TDSEVs to serve diverse biological purposes [105]. Through non-canonical Wnt signaling, colorectal cancer cell-derived SEV Wnt1 can increase colorectal cancer cell proliferation and migration [71]. Wnt5a promotes the aggressiveness of melanoma by stimulating the release of SEVs, enhancing the risk of metastasis [106]. Under hypoxia, colorectal cancer cell-derived SEV Wnt4 promotes the Wnt/β-catenin cascade in endothelial cells initiated by nuclear translocation of β-catenin [107]. Based on these observations, it can bepostulated that Wnt ligands found in TDSEVs could be effective therapeutic agents for controlling EMT induction in cancer cells.

Another signaling pathway implicated in tumor progression is the MAPK signaling system, which regulates cell migration, and apoptosis. The ERK protein subfamily is well-known among the MAPK family for its contributions to EMT [108]. SEVs originating from oral squamous cell carcinoma cells suffering from hypoxia contain miR-21, one of the most dramatically elevated miRs in hypoxic setting conditions, which increases expressions of SNAIL and vimentin while decreasing expression of E-cadherin in oral squamous cell carcinoma cells [109]. SEVs produced from the highly metastatic MHCC97H cells have been shown to augment the motility, chemotaxic ability, and invasion of otherwise less-metastatic cells by initiating EMT through MAPK/ERK cascade [69]. SEV miR-31-5p from hypoxic lung adenocarcinoma cells targets specific AT-rich sequence-binding protein 2 and promotes EMT by trigerring MAPK/ERK signaling [96].

Finally, TGF-β has recently gained increased attention as an EMT inducer [97]. Smad can activate transcription factors such as SNAIL1/2, ZEB1/2, and Twist1, leading to losing of cell–cell adhesion and an increment of mesenchymal markers [110]. Lin et al. [111] utilized TGF-β to induce EMT in Hep3B cells and discovered that SEV miR-374a-5p increased cell proliferation, migration, and invasion. TGF-β1, as reported by Yao et al. [112] induces EMT in endometrial epithelial cells. However, endometrial epithelial cell-derived SEVs can counteract this process. It has been demonstrated that SEVs produced from TGF-β1-treated A549 cells promoted EMT, which was associated with a large enrichment of miR-23a in SEVs [113]. TGF-β, on the other hand, is found in SEVs generated from numerous tumor cells. TGF- β1 is found in SEVs obtained from embryonic cells and mesenchymal stem cells, while TGF-β2 is found in SEVs obtained from ovarian cancer and prostate cancer cells [62]. The findings suggest that TGF-β may potentiate the development of EMT.

### Immune modulation

SEVs can alter the immune system to exert both beneficial and detrimental effects against cancer. TDSEVs bind to immune cells e.g. T cells, B cells, NK cells, monocytes, macrophages, DC, and myeloid-derived suppressor cells (MDSC) and disrupt their anti-tumor abilities [114]. TDSEVs that carry antigens or antigen MHC complexes may directly transfer these components to antigen presenting cells, increasing the ability of displaying TDSEV-derived tumor antigens on their MHC-I/CD8 or MHC-II/CD4 complexes, and thus controlling tumor-mediated T cell responses [115]. TDSEVs, on the other hand, can potentially depress effector T cells by releasing inhibitory molecules, such as programmed cell death ligand-1 (PD-L1) or Fas-ligand (FasL), which reduce activation and/or proliferation of T cells, and may induce death of T cells [116, 117]. PD-L1 mRNA expression in plasma-derived SEVs has been linked to anti-PD-1 therapeutic reaction in melanoma and non-small-cell lung cancer patients [118]. TDSEVs expressing MHC-I-related NK group 2D receptors interact with killer cell immunoglobulin-like receptor 2D ligands available on NK cells, thereby inhibiting the effector function of immune cells in both innate and adaptive immune systems [114]. TDSEVs inhibit monocyte immune function by targeting STAT3 cascade, and upregulating production of arginase and ROS [119]. TDSEVs can polarize M0 macrophages to an M2 phenotype in the lung tumor microenvironment by modifying their transcriptional and bioenergetic profiles [120].

Immunosurveillance represents a pivotal host defense mechanism against the expansion of circulating clones. Notwithstanding this protective mechanism, frequent metastatic events persist, underscoring neoplastic cells' adeptness in evading immune surveillance. Particularly for cancer types disseminating via hematogenous routes, the capability of malignant cells to persist within the bloodstream while eluding immune recognition becomes a critical feature. SEVs, a subpopulation of EVs released by platelets contain P-selectin and GP IIb-IIIa, that have been observed to engage with endothelial cells, cancer cells, and leukocytes [121, 122]. The association between cancer and platelets can be seen as a perilous partnership, whereby platelets act as escorts for cancer cells, accompanying them during circulation and promoting attachment to the vessel endothelium through P-selectin. This process ultimately leads to the migration of cancerous cells to the pre-metastatic niche [123]. Platelets act as protectors of neoplastic cells from immune cell activity, specifically, the antibody-dependent cytotoxicity mediated by NK cells [124]. The process of covering cancer cells with fibrin seems as the primary mechanism attributible for inhibiting the function of NK cells, as it results in impaired recognition of the coated cells. An alternative mechanism involves the secretion of TGF-β1 by tumor cells that are experiencing hypoxia, thereby inhibiting the expression of the activating receptor (NKG2D) of NK cells. The process elicits enhanced activation of the TGF-β1 pathway associated with EMT, thus playing a pivotal role in facilitating the metastatic cascade [125].

**Fig. 4:**
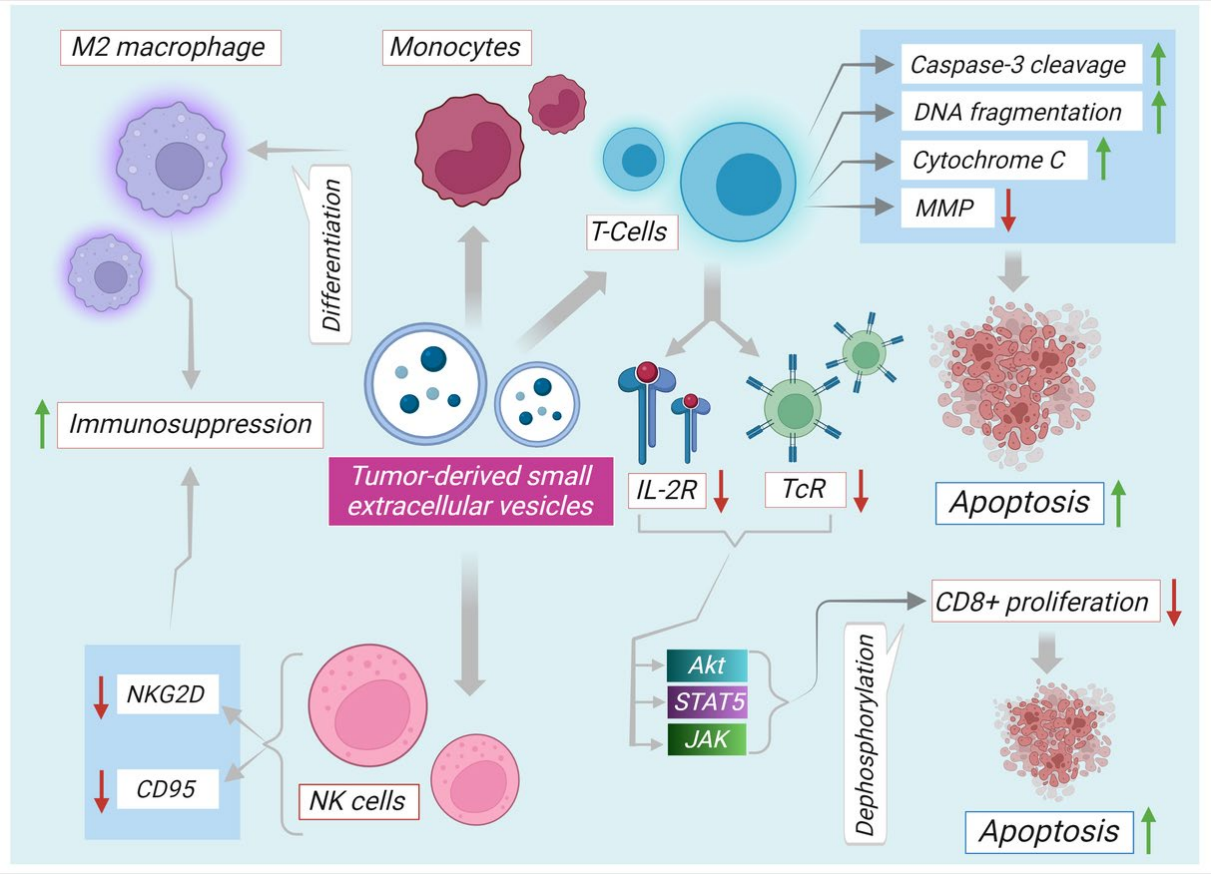
Immune modulatory pathways through TDSEVs. TDSEVs exert detrimental effects on TcR and IL-2R activities. TDSEVs can reduce JAK expression and phosphorylation in activated T cells. TDSEVs can also boost CD4 + T cell expression while lowering CD8 + T cell proliferation. TDSEVs, once again, can increase STAT5 phosphorylation in activated CD4 + T cells while decreasing STAT5 phosphorylation in active CD8 + T cells. TDSEV-mediated apoptosis is characterized by DNA fragmentation, caspase-3 cleavage, mitochondrial cytochrome C release, and MMP loss. TDSEVs also impact the PI3K/Akt signaling pathway to increase apoptosis via Akt dephosphorylation. TDSEVs can also excite NK cells by activating NKG2D receptors on the surface of SEVs. Interestingly, cancer cell growth, invasion, and migration can be aided by M2 macrophage ‘↑’ indicates upregulation, ‘↓’ indicates downregulation. Arrows indicate downstream cellular events/activation. IL-2R. interleukin 2 receptor; MMP. Mitochondrial membrane potential TcR, T cell receptor

On arrival at the secondary organ site, TDSEVs initiate intricate immunosuppressive mechanisms, strategically mitigating the inhospitable microenvironment and fostering the proliferation of cancerous cells. This result is achieved by curtailing the functionality of effector cells and inducing the activation of regulatory T cells [126]. The potential immunosuppressive properties of TDSEVs may be attributed to miRs, as demonstrated in studies on nasopharyngeal cancer [127, 128]. The study outcomes revealed the presence of immunosuppressive miRs (hsa-miR-24-3p, hsa-miR-891a, hsa-miR-106a-5p, hsa-miR-20a-5p, and hsa-miR-1908) within TDSEVs. The hypothesized routes of T cell suppression involve the production of extracellular adenosine through TDSEVs expressing CD39 and CD73 [129]. This event could arise from epigenetic alterations induced by TDSEVs within T cells [129]. Additionally, TDSEVs display the potential to reduce the expression of genes linked to regulatory T cells, subsequently impacting the adenosine pathway, thereby culminating in elevated CD39 expression and heightened adenosine production [130]. TDSEVs employ various immunosuppressive mechanisms to promote the persistence of neoplastic cells. In addition to its role in regulating cellular immune responses, the humoral response, specifically antibody-mediated cytotoxicity, is also affected by TDSEVs. This is noteworthy as antibody-dependent cytotoxicity is one of the major responsive mechanisms, alongside complement-mediated cytotoxicity, that is impacted by TDSEVs. TAMs can be activated through NF-κB and interaction with toll-like receptors located on the surface of macrophage cells [131]. SEV miRs (miR21, miR29a, etc.) can potentially bind to toll-like receptors (TLR-7 and TLR-8) by paracrine manner, leading to a pro-metastatic inflammatory response that involves cytokines TNF-α and IL-6. The association between TDSEVs and inflammatory processes becomes evident in the context of pancreatic ductal adenocarcinoma. Interestingly, uptake of TDSEVs by Kupffer cells of the liver triggers an upsurge in hepatic stellate cell-produced fibronectin synthesis. This phenomenon is facilitated by the action of the macrophage migratory inhibition factor, which augments the secretion of TGF-β1 from Kupffer cells. The hepatic deposition of fibronectin leads to the subsequent immobilization of macrophages derived from bone marrow, thereby establishing a pre-metastatic niche [56]. Immune modulatory pathways affected by TDSEVs are described in Fig. 4. TDSEVs negatively influence the activities of TcR, and IL-2R. TDSEVs can lower the expression and phosphorylation of JAK in activated T cells. TDSEVs have also been found to increase the expression of CD4 + T cells, while decreasing the proliferation of CD8 + T cells. Again, TDSEVs are able to boost phosphorylation of STAT5 in activated CD4 + T cells while inhibiting STAT5 phosphorylation in active CD8 + T cells. TDSEV-mediated apoptosis is characterized by DNA fragmentation, cleavage of caspase-3, release of cytochrome C from mitochondria, and loss of MMP. Akt dephosphorylation is another way that TDSEVs influence the PI3K/Akt signaling cascade to upregulate apoptosis. Furthermore, TDSEVs can activate NKG2D receptors on the surface of SEVs, and stimulate NK cells. Interestingly, cancer cell growth, invasion, and migration can be aided by M2 macrophage polarization as a result of alterations in the tumor microenvironment.

### Pro-angiogenic tumor response

Angiogenesis is a multi-step, complex process that is necessary for the growth, survival, and metastasis of tumors [132]. SEVs assume a pivotal function as intermediaries coordinating interactions between neoplastic cells and vascular cells during hypoxia-initiated pro-angiogenic responses, particularly in hypoxic environments. Moreover, the release of SEVs from melanoma cells fuel a signaling pathway that stimulates the formation of novel blood vessels. This phenomenon is supported by the emergence of endothelial spheroids. The growth of these spheroids is dependent upon the concentration of TDSEVs [133]. The overexpression of Wnt5a in melanoma has a positive correlation with the angiogenic marker ESAM during gene analysis impacting the degree of branching in endothelial cells [106]. SEVs harboring delta-like ligand 4 (DII4) were observed to induce a decrease in filopodia formation within capillary endothelial tip cells and impede the generation of sprouts. These findings suggest that SEVs carrying DII4 can augment the migratory behavior of endothelial cells while concurrently restraining their proliferative activity [134]. A growing body of evidence suggests the involvement of SEV miR in the regulation of angiogenesis and its subsequent effects on the advancement of tumors [135, 136]. For instance, the CD105-positive renal cancer stem cells released pro-angiogenic mRNAs and miRs, which enhanced the development of lung metastases [137]. TDSEVs could transfer miRs from tumor cells to endothelial cells and stimulate angiogenesis [9, 138]. In addition, miR-214 regulated the functioning of endothelial cells and the process of angiogenesis [139]. Transmission of signals between endothelial cells via SEVs, led to the suppression of ataxia telangiectasia mutated (ATM) in the receiving cells. Since ATM is a major factor involved in DNA repair, this may create a promiscuous environment for accumulating genetic mutations and transformation.

Vascular development may be induced by TDSEVs [9]. The most important target of angiogenesis is the VEGF/VEGFR signaling pathway since vascular endothelial cells are essential for angiogenesis and the formation of tumors [140]. TDSEVs induce the production of VEGF by endothelial cells and upregulate VEGFR2 signaling, which leads to an increase in the expression of pro-angiogenic genes and the proliferation of endothelial cells [133]. Hypoxia frequently occurs in tumors and enhances SEV production [141]. TDSEVs generated by hypoxia can form a grid of new blood vessels by modulating regular endothelial cells [142]. This effect may possibly be related to TDSEV-induced loss of E-cadherin and β-catenin from endothelial cell surface, which enhances the mobility of endothelial cells [143]. Endothelial cells secrete cytokines and growth factors which trigger pericytes, multipotent cells that contribute to vascular integrity and regeneration through the PI3K/AKT signaling cascade [144, 145]. SEVs may modulate pericyte functions either to support tumor growth and angiogenesis or to hinder these processes, depending on the specific molecular cargo they carry and the context in which they interact with pericytes [3, 146, 147].

## Oncogenic transformation of healthy cells

In the presence of tumor-derived vesicles, non-neoplastic cells are more susceptible to undergo oncogenic transformations. For example, in an investigation with patient adipose-derived stem cells co-cultured with prostate cancer cells, the former cells experienced a transition from a mesenchymal to an epithelial state and acquired chromosomal abnormalities [148]. Numerous SEVs containing oncogenic agents like miR-125B, miR-130b, miR-155, H-ras, K-ras, Rab1a, Rab1b, and Rab11a were implicated in the adipose-derived stem cells' potential to undergo oncogenic transformation. Furthermore, the suppression of significant tumor suppressor genes such as major tumor suppressor homolog 2 and programmed cell death protein 4 were also involved in this process. This phenomenon is also evident in breast cancer cells. When MCF10A cells were co-cultured with cancer exosomes originating from MDA-MB-231 cells, they underwent neoplastic changes [149]. This was characterized by a decrease in the expression of target transcripts like PTEN and homeobox protein, and a simultaneous increase in the expression of miR-21 and miR-10b. Additionally, oncogenic viruses can induce pro-oncogenic signaling within host cells through the utilization of the EV machinery. Nasopharyngeal cancer cells positive for Epstein-Barr virus have been found to secrete SEVs that contain Epstein-Barr virus oncoproteins, such as latent membrane protein 1 and viral miRs [150, 151]. These exosomes have been observed to promote the expression of AKT, ERK, and EGFR in normal epithelial cells.

## TDSEVs in organ-specific cancer metastasis

A growing body of evidence suggests that TDSEVs can acquire pro-vasculogenic properties [7, 152]. This is caused by an increase in pro-inflammatory molecules and vascular leakiness at the metastatic location. SEVs, which have been aptly termed organ-seeking vesicles, represent a biodistribution that correlates with the pattern of organotropic metastatic dissemination [153].

Pulmonary involvements are common in cancer metastases, and therefore critically important to halt the growth of tumor cells during the colonization of pulmonary metastases [154, 155]. Integrin αvβ5 is key for the progression of pulmonary vascular permeability, especially the β5 subunit, that primarily operates on thrombin, VEGF, and TGF-β. SEV αvβ5 can enhance the migration of gastric cancer cells to lung metastasis [87]. αvβ3 which was first discovered in prostate cancer cell-derived SEVs upregulates tumor metastasis to lungs and bones [86]. TDSEVs also promote tumorigenesis in lung cells through other mechanisms. Upon exposure to cigarette smoking extract, bronchial epithelial cells exhibited an augmentation in SEVs carrying miR-21, which was facilitated through the activation of the signal transducers and activators of the transcription 3 (STAT3) signaling pathway. The presence of SEV miR-21 contributed to the increase in VEGF, thereby favoring tumor angiogenesis, as well as malignant transition of bronchial epithelial cells [156, 157]. Furthermore, miR-21 released by human bronchial epithelial cells (HBECs) enhanced the proliferation of adjacent normal HBECs, supporting the idea of exosomal miRs playing a role in intercellular communication during the course of carcinogenesis triggered by environmental toxins [158]. In a relevant study, TDSEVs were found to increase the mRNA expressions of TLR-2, TLR-7, and TLR-8 in MSCs, particularly TLR-2. The increased TLRs, in turn, stimulated the production of inflammatory substances and the expression of Hsp70, consequently leading to the activation of the NF-κB signaling pcascade stimulating the growth of lung cancer cells [159]. Exosomal miR-23a can accelerate the progress of lung cancer by decreasing prolyl hydroxylase 1/2 (PDH1/2) and accumulating hypoxia-inducible factor-1 (HIF-1) under normoxic as well as hypoxic conditions. Exosomal miR-23a, in addition hindered the tight junction protein Zonula occludens-1 (ZO-1), thus enhancing vascular permeability and promoting transendothelial migration of cancer cells [160, 161]. Through binding with the 3′-untranslated region of wild-type LIM-domain only protein 7, circulating SEV miR-96 increase lung cancer growth. Consequently, the miR-96-LMO7 axis emerges as a potential therapeutic target to treat lung cancer, offering opportunities for novel diagnostic and therapeutic approaches [162]. Notably, it has been proposed that CD105 + microvesicles discharged by SCID mice bolstered the development of pre-metastatic niche within the lungs. Moreover, CD105 + Microvesicles were correlated with increased expressions of MMP2, MMP9, and VEGFR1 levels [137]. Overexpression of small GTPase and Rab3D may upregulate lung metastasis by triggering EMT via intracellular AKT/GSK-3β signaling and boosting the expression of Hsp90, ultimately enhancing cancer metastasis by activation of MMP2 [163].

Melanoma, lung cancer, and breast cancer patients are the most likely to initiate brain metastases. Although the frequency of brain metastasis should decrease as primary tumor detection and therapy improves, but the overall median survival time following diagnosis with current treatment regimens is still less than one year [164]. The brain is often regarded as a "sanctuary site," safeguarded by the blood–brain barrier (BBB) which shields it from infiltrating tumor cells and restricts the entry of several systemic therapeutic agents. Consequently, the capacity of tumor cells to survive within the brain parenchyma post-BBB traversal fundamentally influences the development of metastasis. Numerous factors, such as secreted proteins and miRNAs contained within TDSEVs have been identified for their role in promoting the survival and proliferation of brain metastases. [165–167]. Transport of cancerous cells beyond BBB involves the proteolytic breakdown of the junctional adhesion molecule B (JAMB-JAM2) by cysteine cathepsin S released from tumor cells [168]. Although the significance of this protease in TDSEVs is unknown, it has been found in microglia-derived SEVs, supporting the notion that SEVs produced by brain cells might play a role in BBB disruption. TDSEVs can impair BBB permeability, and breast cancer-derived exosomes can essentially transfer miR105 to endothelial cells to affect the tight junctions [169]. MiR105 translocation increases vascular permeability while decreasing ZO1 expression, resulting in BBB rupture and lung and brain metastases [169]. Similarly, miR-181c in breast cancer-derived EVs triggers BBB breakdown by aberrant actin localization caused by endothelial cell downregulation of its target gene phosphoinositide-dependent kinase-1 (PDPK1) [170]. Through a mechanism involving miR-181c, brain metastasis can be selectively reinforced by breast cancer-derived EVs [170]. Because of inhibition of pyruvate kinase, high amounts of miR-122 released by breast cancer cells limit glucose uptake by niche cells [171]. As a result, breast tumor cells get ample opportunity to adapt to the microenvironment in the pre-metastatic niche by enhancing the amount of accessible glucose. In vivo suppression of miR-122 can restore glucose absorption in distant organs like brain and lungs, reducing metastases [171]. Astrocytes and stromal cells aid breast cancer spread to the brain by turning off PTEN in the cancer cells [172]. Treatment with astrocyte-derived exosomes, on the other hand, results in a dose-dependent rise in miR-19a followed by a decrease in PTEN mRNA expression by brain metastatic breast cancer cells [173].

Exosomes generated from brain metastasized cells have been shown to exhibit common signatures. Exosomes generated from brain metastatic breast cancer cells upregulated miR-210 and downregulated miR-19/miR-29c [174]. Similarly, proteins involved in cell to cell communication, cell cycle, and important cancer metastasis and invasion pathways were enriched, albeit the importance of these molecules in brain metastasis remains unknown due to a lack of verified functional investigations in vivo [175]. ITG3 was found in a collection of exosomes derived from brain metastatic breast cancer and melanoma models using quantitative mass spectrometry [176]. Considering the comprehensive array of data, the disruption of the BBB triggered by TDSEVs is the initial hallmark in the establishment of a pre-metastatic niche within the brain. This process, along with the ensuing intercellular exchange of miRs between tumor and stromal cells, likely constitutes the fundamental mechanisms for EVs to facilitate brain metastasis. In upcoming days, investigations are poised to investigate the involvement of various cell types, such as microglia or immune cells, in the selection and formation of brain metastatic clones, as well as the identification of brain-specific receptors present within EVs.

Cancers of breast and prostate display an undeniable proclivity to colonize bone as a secondary site, with occurence rates of 70 and 90%, respectively [177]. While the importance of TDSEVs with regard to pathogenesis and spread of cancer has been well established, their function in bone metastases remains largely unknown. To date, the findings indicate that TDSEVs can direct bone cell behavior towards a milieu that promotes tumor cell homing [178]. Consistent with their proclivity to induce osteolytic metastases, lung cancer-derived EVs enhance osteoclastogenesis via an exosome-mediated transfer of Amphiregulin [179]. Furthermore, treatment of bone marrow-derived monocytes with TDSEVs enhances osteoclast formation via shuttling miR-21, suppressing programmed cell death 4 (Pdcd4), an osteoclastogenesis-related tumor-suppressing transcription factor [180]. Different integrins play an important role in the bone metastasis of cancer. For example, α4β1 and αvβ3 may increase tumor cell proliferation and metastasis to the bone microenvironment. This effect could be explained by their interplay with VCAM-1 found in bone marrow stromal cells [62]. Integrin β3 is essential for proper workability of newly generated blood vessels in the bone marrow, primary purpose of it being to form the isoforms α2bβ3 and αvβ3 by heterodimerization of respective subunits. α2bβ3, and αvβ3 are critical for entrapping melanoma cells within bone capillaries [181, 182]. Furthermore, the β3 subunit has been found in SEVs derived from melanoma, ovarian cancer and prostate cancer cells [70].

The bone microenvironment is composed of numerous components that work together to sustain bone homeostasis. They include immune cells generated from bone marrow, osteoblasts, osteoclasts, stromal cells, fibroblasts, and a variety of cytokines and growth hormones [183–185]. ECM remodels similarly to bone resorption, especially during breast cancer bone metastases (BCBM). Any changes in the tumor microenvironment add pressure and compression, causing increased rigidity of the bone matrix, which induces a detrimental phenotype in tumor cells by regulating the expression of bone resorption genes [186]. The equilibrium between osteoclast-mediated bone resorption and osteoblast-mediated bone creation is maintained in healthy settings. Tumor cells, on the other hand, imitate bone cells and disturb bone rebuilding. By secreting cytokines and growth factors such as interleukins, TNF-α, VEGF, and macrophage colony-stimulating factor (M-CSF), metastatic tumor cells directly increase osteoclast activity. Additionally, these cells enhance osteoclastogenesis by indirectly stimulating osteoblasts to release RANKL and M-CSF [187]. The interaction of TDSEVs and indigenous bone cells in the bone microenvironment can significantly influence metastasis. Many investigations have been conducted to determine how TDSEVs affect osteoblasts and osteoclasts during bone metastasis both in vitro and in vivo [188, 189]. Breast cancer cells release an increased quantity of SEVs. MDA-MB-231-derived EVs lowered the number, metabolic activity, and alkaline phosphatase activity of osteoblasts. In addition, MDA-MB-231-derived EVs also reduced the transcription of cyclin D1 and differentiation genes in osteoblasts while increasing the expression of pre-osteoclastic proteins including IL-6, RANKL, and others [190]. Breast cancer-derived EVs have been found to reduce MSCs' ability to develop into osteoblasts. They also reduce osteoblast-mediated type I collagen production, which is necessary for bone growth [191]. Likewise, the progression of prostate cancer-induced bone metastasis involves prostate cancer cells reorganizing interactions with sorrounding stromal cells through SEV communication. Consequently, the impacted stromal cells undertake alterations that enhance the microenvironment, facilitating tumor growth and the metastatic process. [192]. Experimentally, it has been confirmed that the content of miR-940 in the SEVs of patients with prostate cancer that has metastasized to the bone is significantly higher compared to the content in the SEVs derived from prostate cancer patients that has not metastasized to the bone, and that miR-940 is associated with the modulation of osteoclast and osteoblast functions to upregulate bone metastasis [193]. The in vitro and in vivo loss-of-function tests revealed that SEV miR-95 might boost the proliferation, invasion, and EMT of prostate cancer cells by directly binding to its downstream target genes, thus increasing prostate cancer bone metastases [194]. Interestingly, SEVs not only promote but may also hinder the progression of prostate cancer. The released SEVs have been shown in both in vivo and in vitro trials to reduce prostate cancer cell proliferation, growth, and bone metastasis, by causing apoptosis, whereby miR-145 plays an important role. On deleting miR-145 gene, the inhibitory impact of the SEV is eliminated, and bone metastasis becomes more likely [195]. By the increased expression of miR-143, bone marrow-derived mesenchymal stem cell-derived SEVs might downregulate Trefoil factor 3, reducing the proliferation, migration, and invasion of prostate cancer cells. It may also induce apoptosis in prostate cancer cells and effectively prevent bone metastases [196]. These studies exemplify the potential of specific cancer cells' released SEVs for future cancer therapy and are discussed below.

## TDSEVs in cancer theranostics

Classical cancer therapies have not always been effective and they often fail to cope with the insidious nature of metastasis and recurrence [197, 198]. The specific microenvironment and communication between tumor cells leave an impact on how quickly a tumor develops. SEVs have received a lot of attention in cancer therapy targeting a variety of pathways over the past few years.

### Prevention of exosome formation and secretion of EVs

Tumor cells secrete a large amount of SEVs which aids in tumor metastasis. Considering this, preventing the release of TDSEVs and/or TDEs may be a potential strategy for tumor management [199]. One of the prime molecules to be linked to exosome secretion is neurophospholipase 2 [200]. The neurophospholipase 2-dependent pathway controls the number of exosomes, and upregulating the expression of neurophospholipase 2 stimulates the synthesis of exosomes [201]. Interestingly, TDSEVs aid in the different stages of cancer metastasis and participate in drug resistance. Consequently, the strategic reduction of SEV secretion originating from cancer cells could potentially offer therapeutic benefits for cancer patients. The modulation of SEV trafficking or the pathways involved in exosome biosynthesis stands as the focal point of the presently employed inhibitors. [202]. D-pantethine, neutral sphingomyelinase inhibitor GW4869, and tipifarnib can potentially prevent the production of exosomes [203]. Ras inhibitor manumycin A, cytoskeleton reorganizing Rho-associated protein kinase (ROCK) inhibitor Y27632, and cysteine proteinase inhibitor calpeptin are exosome trafficking inhibitors [204]. Hence, they are attracting growing research interests regarding their potential utilization in the fight against cancer. Future studies need to explore non-toxic concentrations of the molecules to the cells to assure that the effects are not due to mere cellular cytotoxicity, and then due consideration should be given to characterizing EVs. Manumycin A could dramatically reduce exosome secretion in prostate cancer cells by roughly 55%. Additionally, its exosome inhibitory action did not manifest in healthy cells, indicating that it could only suppress the exosomes generated by cancer cells [205]. Calpeptin is among the most well-studied calpain inhibitor at the moment. Calpains enhance shedding of Microvesicles via cytoskeletal reorganization; hence, calpain inhibitors such as calpeptin can diminish MV shedding by prostate cancer cells while also reducing cell proliferation [206]. Y27632 is a competitive inhibitor of ROCK1 and ROCK2, two ROCK family members that interact with the cytoskeleton [204]. Y27632 competes with ATP for the catalytic binding sites on ROCK1 and ROCK2. Y27632 has a strong effect on Microvesicles, reducing their production by 67% in PC3 cells [207]. Interestingly, TDSEVs expressed a high level of PD-L1, which led to T-cell exhaustion, and the ensuing tumor resistance to immune checkpoint inhibitors. Rab27a and neutral sphingomyelinase-2 (nSMase2) was deleted using the CRISPR/Cas9 gene-editing approach. Both deletions were shown to lower exosomal-PD-L1, while the loss of nSMase2 resulted in lower levels of cellular and extracellular PD-L1, but not of cell-surface PD-L1 [208]. Production of exosomes can also be downregulated by inhibiting the enzyme nSMase using GW4869 [209]. One of the most potent nSMase2 inhibitor reported so far is DPTIP, which inhibits exosome secretion in a dose-dependent manner [210]. It has been demonstrated that ferroptosis inducer and GW4869 effectively blocked SEV PD-L1-regulated immunosuppression, restored anti-tumor immune response, and decreased metastasis in melanoma model when exosomes were eliminated [211]. A study by Datta and peers [212] focused on the inhibitory effects of tipifarnib on the biogenesis of exosomes in aggressive prostate tumor cells by inhibiting the expressions of Rab27A, Alix, and nSMase2; which also mitigated metastasis of cancer cells. Further, the inhibitory effect of tipifarnib is selective to cancer cells only, since it affects the release of exosomes in C4-2B and PC-3 malignant cells while sparing human prostate epithelial cells [213]. This selectivity makes it one of the forerunners for probable clinical utilization. Research also revealed neticonazole and climbazole, two anticancer agents that can also inhibit EV release by similar mechanisms, and arrest cancer metastasis; however, their efficacy seemed inferior to tipifarnib [212].

EV secretion can be indirectly inhibited by proton-pump inhibitors. Of note, these inhibitors have been found to inhibit exosome release while also promoting the retention of chemotherapeutic drugs inside tumor cells [214]. Thus, they potentially offer dual benefit in cancer therapeutics. Extracorporeal hemofiltration could be employed in addition to proton-pump inhibitors to get rid of SEVs generated from circulating tumors [215]. A recent study uncovered the anticancer and anti-metastatic potential of apatinib, highlighting the downregulation of proteins such as Rab11, vesicle-associated membrane protein (VAMP), Snap23 (regulatory proteins for multivesicular body transport) to impart inhibitory effects on exosome secretion in metastatic colorectal cancer [216]. Cannabidiol, a phytocannabinoid can selectively inhibit SEV release from cancer cells exerting an effect against tumor metastasis [217, 218]. The expression of CD63 dramatically decreased in cancer cells (HepG2, MDA-MB-231 and PC3) after receiving 1 h of cannabidiol treatment, suggesting that the underlying mechanism involves its interference with CD63 [217]. Glyburide (glibenclamide) and indomethacin inhibits ATP-binding cassette (ABC) transporter. Glyburide blocks ATP-sensitive K^+^ channel of an ABC transporter that is involved in the secretion of EVs [204]. On the other hand, the anti-inflammatory medicine indomethacin selectively inhibits the transcription of ABCA3, to help in the transport of lipids [219]. U0126 inhibits MEK 1 and MEK 2, and MAPK, preventing ERK activation, which is required for microvesiculation to occur [204]. U0126, on the other hand inhibits SEV secretion and, when combined with gemcitabine for 72 h, accelerated cell mortality in chemoresistant Suit-2 cells by 11-fold compared to the control [146]. Imatinib and dasatinib inhibit the ATP-binding sites of the catalytic sites of bcr-abl tyrosine kinase enzymes. In cancer-derived SEVs, phosphorylated receptor tyrosine kinases increase anti-apoptotic activity in monocytes [220]. Dynasore is a well-known clathrin-dependent endocytosis (CDE) inhibitor that has been intensively studied. However, dynasore, like the other CDE inhibitors, may have non-specific effects. Dynasore suppresses GTPase activity of the dynamin proteins dynamin1, dynamin2, and Drp1 (mitochondrial dynamin) non-competitively in seconds. Dynamin proteins are required for a late stage of CDE that requires the formation of a clathrin-coated endocytic vesicle and may also be involved in early stages of CDE. Through these mechanisms, dynasore inhibit endocytosis of SEVs [221]. Genistein inhibits tyrosine kinases, including the EGF receptor kinase, in a highly selective and dose-dependent manner [222]. It disrupts actin configurations and hinders dynamin mobilization for plasma membranes, both of which are required for clathrin-independent endocytosis [222]. Thus, exosome inhibitors and EV secretion inhibitors seem to offer potential therapeutic candidates for clinical translation against cancer. Table 1 represents an extensive list of exosome inhibitors and EV inhibitors on the basis of mechanism-based classification. Interestingly, few agents can act by more than one mechanisms. Repurposing of medicines also is gaining attraction in this regard. Quite clearly, better understanding of the mechanism of SEVs release would lead to more efforts towards development of therapeutically useful SEV inhibitors as adjunctive therapy for cancer.

**Table 1:** Mechanism-based classification of SEV inhibitors

Mechanisms of action	Inhibitors
Sphingomyelinase inhibitors	GW4869
	Cambinol
	Spiroepoxide
	Imipramine
	DPTIP
	Manumycin A
ABC transporter inhibitors	Glyburide
	Indomethacin
Phosphatidylserine translocation inhibitors	D-pantethine
	Bisindolylmaleimide-l
Endosomal membrane inhibitors	Simvastatin
Cytoskeleton-related protein inhibitors	Calpeptin
	Chloramidine (Cl-amidine)
	NSC23766
	Y27632
	Cytochalasin D
Protein kinase inhibitors	Y27632
	U0126
	Imatinib
	Dasatinib
ESCRT-pathway inhibitors	Manumycin A
	Tipifarnib
	Sulphisoxazole
	Apatinib
EV release inhibitors	Cannabidiol
	SMR peptides
	Ketotifen
	Dimethyl amiloride
Endocytosis inhibitors	Dynasore
	Ikarugamycin
	Genistein
	Chlorpromazine
	MβCD
	Heparin
	ΕΙΡΑ

### Biomarkers

The scrutiny and full characterization of TDSEVs could substantially advance the early detection of tumors and introduce novel approaches for tumor management. The multifaceted composition of TDSEVs, with molecules like miRs, lncRNAs, circRNAs, and mRNAs, can be used to diagnose tumors at an early stage and/or to monitor the progression of established tumors. SEVs function as efficient carriers of diverse cargo, particularly miRs, which offer considerable potential as prognostic biomarkers. The distinctive alteration of miR-expression patterns by tumors generates distinct profiles that differentiate them from healthy cells, rendering these miRs valuable tools for prognosis [223]. In the recipient cells, miRs can attach to specific mRNA sequences and hinder the process of translation. It is a well-established fact that miRs can modulate the expression of over 50% of the proteins that are encoded by genes [224]. MiRs have a crucial function in the regulation of receptor cells by binding to the specific 3′ untranslated region (UTR) of the target genes. This binding leads to the transcriptional suppression of these target genes [225]. It has been observed that the dysregulation of miR expression is a common occurrence in many types of cancers [226]. Extensive research has been conducted on biomarkers in patients with prostatic carcinoma. Following radical prostatectomy, SEV miR-141, and miR-375 have been found to be present in significantly higher concentrations in prostate cancer patients [227, 228]. In addition, differential expression of miRs has the potential to serve as a diagnostic tool for discriminating between high and low-grade tumors, thereby facilitating timely risk stratification. The combination of miRs has the potential to serve as a diagnostic tool for colorectal cancer [229]. Dou et al. [230] revealed that tumor cells and extracellular microvesicles harbor both miRs and circRNAs. The presence of circRNAs was found to significantly decrease the expression levels in cells with KRAS mutations. The levels of circRNAs in tumor tissues of cancer patients were observed to be significantly downregulated incomparison to healthy counterparts. This suggests a potential association between circRNAs and the onset and progression of tumors. SEV AR-V7 can serve as a valuable biomarker for predicting the effectiveness of hormone therapy in prostate cancer patients, as demonstrated by the positive results of the median progression-free survival analysis and overall survival analysis [231]. According to a clinical laboratory report, the utilization of both SEV RNA and cell-free DNA techniques for detection exhibits greater sensitivity in detection of EGFR mutations in plasma in patients detected with non-small cell lung carcinoma, compared to the use of cell-free DNA alone [232]. The concomitant identification of SEV RNA and cell-free DNA enhances the efficacy of detecting EGFR mutations in plasma, thereby establishing TDSEVs as viable biomarkers in cancer diagnostics. Increased miR-21 levels in circulating SEVs have been linked to a variety of cancers, including breast, colorectal, esophageal, gastric, liver and ovarian cancer, and an elevated level of SEV miR-21 produced from urine has been linked to bladdercancer and prostate cancer [233]. SEV miRs have been studied for their capacity to predict patients' therapy response and outcome for a diverse range of cancers, adding to early detection and prognosis. SEV miR-146a-5p levels were found to be a strong predictor of cisplatin response, while SEV miR-425-3p and miR-96 levels could predict cisplatin resistance in lung cancer [234, 235]. Furthermore, high miR-155 and miR-301 quantities in circulating SEVs have been linked to complete response to neoadjuvant therapy in breast cancer patients [236]. Elevated levels of miR-155, together with miR-301 and miR-339-5p in serum SEVs, have been shown to predict gemcitabine resistance in pancreatic ductal adenocarcinoma, sensitivity to neoadjuvant therapy in breast cancer, and pre-operative radiation in locally advanced esophageal squamous cell carcinoma [237]. Furthermore, serum exosomal miR-718 was found to be adversely associated with the recurrence rate in hepatocellular carcinoma [238]. By incorporating a reporter mRNA, Skog and colleagues [239] have revealed that EVs from glioblastoma cells carrying mRNAs and miRNAs can act as prometastatic agents, and these RNA fragments can be translated at the recipient cells. These EVs, found in the serum of glioblastoma patients thus can serve as biomarkers for glioblastoma and its metastasis [239]. Again, miR451a has been linked with metastasis of lung cancer cells to lymph nodes [240]. On a similar note, miR222 is connected to lymphatic metastasis in highly expressed breast cancer [241]. Since many lncRNAs have tissue-specific expression, analyzing SEV lncRNAs may also be a reasonable approach for cancer diagnosis. In hepatocellular carcinoma, for example, lncRNA-ATB was discovered to be a predictive marker when combined with miR-21 [242, 243]. Similarly, serum SEV lncRNA-UCA1 and HOTTIP have been identified as promising biomarkers for bladder and gastric cancer, respectively [244, 245].

SEV protein markers also exhibit promise as biomarkers. Exosomes containing glypican-1 have been revealed to be a sensitive and specific diagnostic markers for pancreatic cancer [246]. In addition, there is a correlation between the concentration of glypican-1 and tumor load, thereby enabling timely identification of relapse after surgery or metastatic dissemination. Although the findings are noteworthy, the small sample size indicates the necessity for conducting more extensive studies to validate these conclusions. In addition to pancreatic cancer, the aforementioned study demonstrated an increase in glypican-1-containing exosomes in individuals with breast cancer as compared to healthy individuals [246]. The involvement of SEV transmembrane protein 256 has been suggested in individuals diagnosed with prostate cancer, exhibiting a considerable degree of efficacy in both sensitivity and specificity [247]. Growing body of evidence indicates that SEVs may also serve as innovative diagnostic biomarkers for various types of cancer [248, 249]. SEVs derived from the serum of cancer patients have been found to play a significant role in enhancing the invasiveness of tumors [250]. However, knowledge about their impact on the survival and proliferation of tumor cells is limited. The levels of expression of TDSEVs have been observed to be significantly elevated in comparison to those of healthy individuals. This observation has further been corroborated through experimental validation.

An analytical approach has been devised for profiling circulating EVs directly from blood samples of colorectal cancer patients [251]. Cancer-derived EVs captured by two types of antibodies are detected with photosensitizer-beads without necessitating a step for purification. Similarly, circulating EVs can also be used to detect colorectal cancer using antigen CD147, embedded in cancer-linked EVs. The work by Yoshioka and peers [251] presents insights in translational medicine from diagnostic as well as therapeutic perspectives, while introducing a novel liquid biopsy approach to sensitively identify disease-specific circulating EVs. Notably, the SEV double stranded DNA has been revealed to reflect the full genome and represent the parental tumor cells' mutational state [252]. In the preponderance of ovarian cancer patients' circulating vesicles, the expression of claudin proteins associated with ovarian cancer has been detected [253]. Growing evidence suggests that, unlike non-tumorigenic cells, tumor cells manyatimes reveal phosphatidylserine onthe surface [254–256]. An ELISA-based technique for detecting picogram levels of SEV phospholipid in plasma as a cancer biomarker has been designed, capable enough to distinguish breast cancer-bearing animals and normal controls [257]. A similar study discovered that individuals with ovarian cancer had considerably higher quantities of phosphatidylserine-positive SEVs than healthy controls [254] Table 2 enlists the SEV-derived components that have come up as potential biomarkers for the diagnosis and prognosis of various types of cancers.

**Table 2:** TDSEV-derived components as prospective biomarkers for the diagnosis and prognosis of different types of cancer

S. No	Types of cancer	SEV components	Diagnostic/Prognostic significance	References
1	Breast cancer	miR-223-3p	Biomarker for early detection of breast metastasis	[258]
2	Breast cancer	miR-222	It is connected to lymphatic metastasis in highly expressed breast cancer	[241]
3	Lung cancer	miR-106b	It is extensively expressed in serum and has been linked to lymph node metastasis as well as MMP protein expiration in lung cancer metastasis	[259]
4	Lung cancer	miR-451a	It aids in the spread of lung cancer to lymph nodes	[240]
5	Colon cancer	CD147	It is substantially expressed in patients with colon cancer	[260]
6	Colon cancer	miR-203	It is significantly expressed in colon cancer and is linked to liver metastasis	[261]
7	Prostate cancer	miR-501-3p	It is reduced in prostate tumors, yet it lowers E-cadherin expression and boosts metastasis	[262]
8	Prostate cancer	miR-1290 and miR-375	These are significantly expressed in prostate cancer and are linked to poor overall survival in castration-resistant patients	[263]
9	Liver cancer	circRNA- 100,338	It promotes the metastasis of liver cancer	[264]
10	Liver cancer	miR-126	It is a useful prognostic biomarker for liver cancer	[265]

### TDSEVs as carriers

SEVs are recognized for their safety and non-toxicity in human tissues due to their biocompatibility and membrane-bound composition [266, 267]. The presence of different integrins on the surface of TDSEVs enables them with the capability to engage adhesion molecules on cell surfaces, thereby enhancing the precision with which targets can be reached [268]. The fundamental benefit of using SEVs comprises their small size, which facilitates efficient drug delivery by enabling them to cross various biological barriers, including the blood–brain barrier (BBB) [269]. Their capacity to engage in physiological communication with cells, coupled with minimal toxicity, enhanced permeability, and immune transparency, underscore the fact that SEVs are highly desirable nanocarriers for drug delivery [270]. TDSEVs remain stable within the circulation and are capable of traversing significant distances within the body, allowing targeted delivery of therapeutic agents to specific tissues or cells [271, 272]. Additionally, since TDSEVs possess strong hydrophilicity, along with a lipophilic membrane, it enables them to transport both hydrophilic and hydrophobic agents [273]. TDSEVs as carriers can boost drug accumulation at the tumor location, get beyond tumor therapy restrictions, and trigger the death of cancer cells [200]. Exosomes and SEVs are superior to other nanoscale drug delivery methods in various ways. They are non-immunogenic by nature and resemble their own cells in vivo at the constitutive level [200]. TDSEVs can overcome biological barriers and minimize cytotoxicity and immunogenicity, whereas major drawbacks of organic and inorganic nanoparticles are toxicity, lack of targetability, and immunogenicity [200]. The variability of cargos that can be loaded into TDSEVs for cancer therapeutics, such as drug molecules, proteins, and ncRNAs, makes SEVs an even more efficient therapeutic tool [274].

Leveraging TDSEVs as carriers, a CRISPR/Cas9 plasmid has been effectively delivered to ovarian cancer cells resulting in the downregulation of PARP-1 expression, sensitizing the tumor cells to cisplatin treatment. The ensuing synergistic cytotoxic effects underscore the potential of exosomes as a promising avenue for tumor therapy [275]. The combination of SEVs’ ability to specifically target tumor cells and TDSEVs' high cell absorption rate can significantly increase delivery and the drug's potential to fight cancer. SEV miR-302b has been proposed as a promising gastric cancer therapeutic since it targets the ERK pathway and decreases cancer cell proliferation and migration [276]. AS1411 aptamer-conjugated extracellular nanovesicles derived from cancer cells exhibited cancer-specific targeting, significantly improving the therapeutic efficacy of paclitaxel [277]. Biologically derived TDSEVs, owing to endogenous origin portray enormous potential to facilitate future clinical adoption. OVA antigen-carrying cancer cell-derived EVs induced OVA-specific CD4^+^ and CD8^+^ T cell-mediated immune response, significantly inhibiting tumor growth [278, 279]. The delivery of EV-based DNA vaccines presents a novel approach for inducing robust CD8^+^ T cell responses to the antigen, thus rendering it a promising candidate for cancer immunization. Table 3 enlists SEV-based therapeutic approaches for cancer.

**Table 3:** SEV-mediated delivery of therapeutic materials as anticancer strategies

S. No	Types of cancers/cells targeted	Targeting ligands	Therapeutic payloads	Effects	References
1	Adenocarcinoma	iRGD peptide (Arg-Gly-Asp peptide)	Kirsten Ras oncogene short interfering RNA	Targets Kirsten Ras oncogene	[280]
2	Breast cancer	*α*v-integrin-specific iRGD peptide	Doxorubicin	Targeted delivery of doxorubicin	[281]
3	Breast cancer	GE11 peptide	miR-let7a	Targets EGFR-expressing tumors	[282]
4	Breast cancer	DARPin (For HER2-positive breast cancer)	Tpd50 siRNA	RNAi treatment of HER2-positive breast cancer	[283]
5	Triple negative breast cancer	Folate	Elastin	Ferroptosis induction in targeted cells	[284]
6	Epithelial carcinoma	Folate	siRNA within folate/SEV complex	Efficient cancer suppression	[285]
7	Breast cancer	SEVs conjugated with AS1411 Aptamer	Paclitaxel (PTX)	Targeted anticancer effects	[286]
8	Breast cancer	*α*CD3/*α*EGFR (for EGFR-positive breast cancer cells)	Smart-exosomes	Cell-free cancer immunotherapy	[287]
9	Triple negative breast cancer	NLS peptide	Photosensitizer	Dual-stage light-guided cell membrane and nucleus-targeted photodynamic treatment	[288]
10	Colorectal cancer	NLS peptide	Photosensitizer	Dual-stage light-guided cell membrane and nucleus-targeted photodynamic treatment	[288]
11	Colorectal cancer	HER affibody	5-fluorouracil, anti-miR-21	Reverses chemoresistance to improve treatment of cancer	[289]
12	Embryonic fibroblasts	mRNA	SIRP*α*	Increased SEV circulation time	[290]
13	Glioma	neuropilin-1-targeted peptide	Curcumin-SPION	Simultaneous diagnosis and therapy of glioma	[291]
14	Glioma	ApoA-1 mimetic peptide	Methotrexate, KLA (Lys-Leu-Ala)	Selective treatment of glioblastoma multiforme	[292]
15	Lung cancer	iRGD peptide (human alveolar basal epithelial cancer cells)	Kirsten Ras oncogene short interfering RNA	Targets Kirsten Ras oncogene	[280]
16	Lung cancer	A linear truncated form of LyP-1 (in nonsmall cell lung carcinoma)	SOX2 siRNA (silencing RNA)	Gene delivery for cancer therapy	[293]
17	Lung cancer	AA symmetrical bidentate ligand	PTX	Improves drug circulation and counteracts pulmonary metastases	[294]
18	Leukemia	IL-3	Imatinib, BCR-ABL siRNA	Inhibits cancer cell development, enhanced intratumoral accumulation	[295]
19	Glioma	Exosomes	Nucleic acids	Tumor suppression, inhibition of tumor growth	[296]
20	A549 stem cells	Linear truncated form of LyP-1	SOX2 siRNA (silencing RNA)	Gene delivery for cancer therapy	[297]
21	T-cells	*α*CD3/*α*EGFR (for EGFR-positive breast cancer cells)	Smart-exosomes	Cell-free cancer immunotherapy	[298]
22	T-cells	OVA antigen	Antigen	Enhances the immunogenicity of cancer vaccines	[278]

## Clinical translation: Perspectives and challenges

TDSEVs, and SEVs separated from malignant ascites have been evaluated for their capacity to elicit an anti-tumor response in patients. Though these strategies seem to be safe apparently, there has been a deficiency of clinical efficiency of SEV-based approaches contary to the promising outcomes of many in vitro and in vivo preclinical studies. A possible explanation for these observations may be related to TDSEVs' potential immunosuppressive properties, direct administration of TDEs/TDSEVs might lead to accelerated tumor growth. The adverse effects of SEV-inhibitors are yet to be broadly studied. Some of the adverse effects of certain drugs (i.e. imatinib, glibenclamide, and indomethacin) are well established while others are not extensively explored. For clinical translation, more extensive pre-clinical studies are required [204]. To overcome the immunosuppression issue, the focus has shifted to TDSEV-loaded dendritic cells and ascites-derived SEVs. While this is an interesting approach, the small number of clinical trials and recruited patients to date apprehends a conclusive evaluation at this time. While it is widely recognized that cancer patients display a twofold elevation in blood SEV levels compared to healthy adults due to the increased SEV production by cancer cells, unfortunately, the utilization of these for biomarkers in clinical applications has been impeded by challenges related to the cost and methodology of SEV isolation. These observations highlight the need for new approaches that can effectively reduce the contamination of concomitantly isolated protein that aggregates with purified membranous particles.

The pro-tumorigenic potential of TDSEVs in cancer patients is reinforced by the observations that in patients with breast or ovarian cancer, the level of circulating SEVs and SEVs with tumor markers is way higher compared to non-malignant persons, and enhances with tumor progression. Furthermore, SEVs separated from the sera of oral cancer or ovarian cancer patients can impede the function of T lymphocytes and induce apoptosis to them [299, 300]. Thus, it has been hypothesized that omitting immunosuppressive TDSEVs from circulation of a cancer patient would enhance the anti-tumor immune response, and defer the spead and progression of cancer [301]. Only a small number of patients benefited clinically from the less-selective Prosorba Column, a plasma filtering device that captures immune complexes including IgG [214] Higher target-specific affinity filtration systems, like adaptive dialysis-like affinity platform technology, are now being developed and appear effective against cancer metastasis. Before using the same in the clinical context, regimen optimization, and efficacy testing will be necessary [214].

The accumulation of TDSEVs in the peripheral circulation may be influenced by a variety of tumor types and, potentially, tumor growth patterns. Thus, cautious clinical interpretation is required when using TDSEVs in cancer management. In clinical setting, Skog and colleagues [239] detected tumor specific EGFRvIII in many patients with glioblastoma, strengtheing their case as biomarkers.As knowledge regarding the origin of TDSEVs continues to advance, there is promising potential for their utilization in clinical contexts. The utilization of TDSEVs as a means of targeted therapies may offer a promising avenue for cancer patients, providing a novel therapeutic alternative regarding personalized therapy. However, the current understanding of SEVs remains relatively limited, and their roles in immunomodulation and promoting tumor cell resistance are yet to be fully elucidated [302]. Furthermore, a comprehensive investigation into the impact of SEVs on secretory cells is warranted for the future.

## Future prospects

SEVs are important in various biological processes such as tumorigenesis, development, invasion, metastasis, the tumor microenvironment, and drug resistance [303]. The process of SEV biogenesis presents a promising avenue for further investigations, with potential implications for the identification of therapeutic agents aimed at enhancing the efficacy of cancer treatment. TDSEVs have the potential to be utilized comprehensively for the timely prediction, clinical assessment of staging, diagnosis, and therapeutic management of malignant neoplasms. Interestingly, a growing body of evidence indicates that TDSEVs can potentially induce either anti-tumorigenic or pro-tumorigenic effects [304]. The observed effects, which may appear contentious, can be attributed to intricate interplays among SEVs, recipient cells, and contextual factors. The potential immunostimulatory or immunosuppressive effects of TDSEVs in cancer patients may be contingent upon both the stage of cancer progression and the immune status of the patient. TDSEVs, being highly similar to their parental cells, have the potential to transfer the harmful impact of tumor cells to the immune system, thereby supporting their survival, proliferation, and metastasis. While TDSEVs exhibit significant promise for application in pharmaceutical development their ability to modulate the immune system poses a significant challenge [305].

The diverse impacts of TDSEVs play vital roles in the progress of cancer via various mechanisms. SEVs are presumed to be biasedly distributed in part due to the integrin repertoire. Integrin β4, for instance, leads metastasis to the lungs, while integrin β5 promotes SEV adherence in the liver [306]. It is possible that other endocytic pathways, such as lipid raft, clathrin, and caveolin-mediated internalization, are also involved in the uptake of TDSEVs into specific recipient cells. Encapsulation efficiency of SEVs including TDSEVs is significantly greater than that of synthesized nanoparticles [307]. According to reports, SEVs combined with nanoparticles can improve the effectiveness of the drug delivery system's encapsulation, opening new possibilities for SEV research in the future [308–310].

SEVs play a significant role in promoting tumor invasion and metastasis through the process of EMT in epithelial cells, which encompasses several mechanisms. TDSEVs are recognized as regulators of immune cell functionality through diverse mechanisms, including the modulation of immune cell behavior. On the contrary, TDSEVs can amplify the activation of the host immune response against tumor cells. SEVs demonstrate the capacity to encapsulate bioactive entities capable of contributing to drug resistance, invasiveness, and metastasis of cancer cells [311]. Hence, they possess the potential to serve as a focused therapeutic approach for combatting tumors.

There are still several inquiries that are yet to be addressed regarding SEVs, including TDSEVs. The combination of SEVs and CRISPR-Cas genome-editing systems presents a promising avenue for further investigation in the field of precision oncology. Can SEVs be integrated and loaded with ew tumoricidal agents simultaneously in a similar manner? The impact of the route of administration on the therapeutic effectiveness of TDSEV-based therapies remains uncertain. Legislatively speaking, there is currently a lack of established guidelines, specifically for therapeutic development related to TDSEV. However, it is anticipated that regulatory and safety prerequisites for pharmaceutical processes and clinical implementation would be inferred from existing legislation on cell-based protocols. Similarly, the logistical concerns about the processing and manufacturing may present a hurdle, owing to the substantial quantity of TDSEVs required to surmount the inadequate therapeutic effectiveness of nanoparticle delivery [312]. The investigation of cancer-derived SEVs that are imperative targets for any therapeutic regimen is of utmost importance. SEVs can be utilized as cell-free therapies in the domains of cancer vaccines and immunotherapy. In the liquid biopsy era, exosomes might prove to be promising biomarkers for diagnostic, prognostic, and predictive therapy response. For instance, the RNA characteristics of exosomes from different cell sources are different, many RNAs are tumor-specific; therefore, TDSEVs can be utilized as diagnostic as well as prognostic biomarkers for diverse types of cancers.

## Conclusion

SEVs are critical components for intercellular communication. They can perform a variety of activities in the tissues owing to their capability to transfer information to other cells. SEVs transport a variety of cargo, including proteins, miR, mRNA, and nucleic acids, all of which can have distinct functions in target cells. TDSEVs are emerging as possible diagnostic and therapeutic tools. SEV miRs and proteins have been recognized as possible biomarkers for cancer diagnosis, prognosis, and therapeutic response prediction. Since TDSEVs constitute just a small fraction of total SEVs in body fluids, high-sensitivity detection is necessary for TDSEV-based cancer diagnostics. New platforms for SEV separation and detection continue to confront obstacles such as limited yield, low specificity and sensitivity, and significant variability of diverse SEV subsets. The identification of single SEVs could improve our understanding of SEVs coming from tumor-derived sources. All these strategies will facilitate the development and replication of SEV-related cancer detection and treatment. On the other hand, advancements in technology for one-step SEV detection devoid of tedious isolation processes would significantly advance SEV-based biomarker discovery. The important components of SEVs for therapeutic delivery must be defined, as must the methods for producing commercial SEVs. As particular substances within SEVs derive from their cells of origin, TDSEVs might potentially serve as significant diagnostic and prognostic biomarkers. Additionally, the utilization of SEVs as direct therapeutic targets and/or tailored drug delivery cargos might soon offer novel therapeutic avenues.

## Data Availability

Not applicable.

## Abbreviations

Akt: Ak strain transforming
Alix: ALG-2-interacting protein X
APC: Antigen presenting cells
ATM: Ataxia telangiectasia mutated
AXIN: Axis inhibition protein
BBB: Blood Brain Barrier
BCBM: Breast cancer bone metastases
CASCs: Cancer-associated stromal cells
CHMP4: Charged Multivesicular Body Protein 4
circRNA: Circular RNA
CRISPR/Cas9: Clustered regularly interspaced short palindromic repeats and CRISPR-associated protein 9
CSF1: Colony-stimulating factor 1
ctDNA: Circulating tumor cell DNA
DARPin: Designed ankyrin repeat protein
Dll: Delta-like ligand
DPTIP: 2,6-Dimethoxy-4-[4-phenyl-5-(2-thienyl)-1H-imidazol-2-yl]phenol
ECM: Extracellular matrix
EEs: Early endosomes
EGFR: Epidermal growth factor receptor
ELISA: Enzyme-linked immunosorbent assay
EMT: Epithelial-mesenchymal transition
ERK: Extracellular signal-regulated kinase
ESAM: Endothelial cell-selective adhesion molecule
ESCRT: Endosomal complexes required for transport
EVs: Extracellular vesicles
Fas-L1: Fas-ligand
GPs: Glycoproteins
GRB2: Growth Factor Receptor-bound protein 2
GSK-3β: Glycogen synthase kinase-3 beta
HBECs: Human bronchial epithelial cells
HB-EGF: Heparin-binding EGF-like growth factor
HER: Human epidermal growth factor receptor
HIF1: Hypoxia-inducible factor-1
Hrs: Hepatocyte growth factor-regulated tyrosine kinase substrate
Hsp: Heat shock protein
ILs: Interleukins
ILVs: Intraluminal vesicles
JAMB-JAM2: Junctional adhesion molecule B
JNK: Jun N-terminal kinase
LEs: Late endosomes
lncRNA: Long noncoding RNA
MAPK: Mitogen-activated protein kinase
M-CSF: Macrophage colony-stimulating factor
MDSCs: Myeloid-derived suppressor cells
MEK: Mitogen-activated protein kinase kinase
MHC: Major histocompatibility complex
miRs: MicroRNAs
MMPs: Matrix metalloproteinases
MSCs: Mesenchymal stem cells
MT1-MMP: membrane type 1 MMP
MVBs: Multivesicular bodies
Microvesicles: Microvesicles
NF-κB: Nuclear factor kappa-light-chain-enhancer of activated B cells
NLS: Nuclear localization signals
nSMase: Sphingomyelinase
OVA: Ovalbumin
PARP-1: Poly (ADP-ribose) polymerase 1
Pdcd4: Programmed cell death 4
PDH1/2: Prolyl hydroxylase ½
PD-L1: Programmed cell death ligand-1
PDPK1: Phosphoinositide-dependent kinase-1
PI3K: Phosphoinositide-3-kinase
PI3P: Phosphatidylinositol 3-phosphate
PSMA: Prostate-specific membrane antigen
PTEN: Phosphatase and tensin homolog
PTPRB: Tyrosine phosphatase receptor type B
Rab: RAS oncogene family
RANKL: Receptor activator of nuclear factor kappa beta
RGD: Arginylglycyl aspartic acid
ROS: Reactive oxygen species
SEVs: Small extracellular vesicles
siRNA: Silencing RNA
SOX2: Sex determining region Y-box 2
STAT3: Signal transducers and activators of the transcription 3
TAM: Tumor-associated macrophages
TAZ: Transcriptional coactivator with PDZ-binding motif
TcR: T-cell receptor
TDEs: Tumor-derived exosomes
TDSEVs: Tumor-derived small extracellular vesicles
TEAD: TEA domain transcription factor
TfR: Transferrin receptor
TGF: Transforming growth factor
TIL: Tumor infiltrating leucocytes
TLRs: Toll-like receptors
TME: Tumor microenvironment
TNBC: Triple negative breast cancer
TNFs: Tumor necrosis factors
TRAIL: Tumor necrosis factor-related apoptosis-inducing ligand
Tsg101: Tumor susceptibility gene 101
UTR: Untranslated region
VAMP: Vesicle-associated membrane protein
VCAM-1: Vascular cell adhesion molecule 1
VEGF: Vascular endothelial growth factor
VEGFR: VEGF receptor
Wnt: Wingless related interation site
YAP: Yes-associated protein
ZEB1: Zinc Finger E-Box Binding Homeobox 1
ZO-1: Zonula occludens-1
